# Child Sexual Abuse as Lifespan Trauma Within the Context of Intimate Partner Violence: Experiences of Caribbean Women

**DOI:** 10.3389/fsoc.2021.623661

**Published:** 2021-05-11

**Authors:** Adele D. Jones

**Affiliations:** The None in Three Centre for the Prevention of Gender-Based Violence, Department of Behavioural and Social Sciences, The University of Huddersfield, Huddersfield, United Kingdom

**Keywords:** child sexual abuse, Caribbean, pregnant, living with HIV, disability, woman, IPV

## Abstract

**Background:** There is a dearth of research which explores sexual abuse from perspectives of Caribbean women, despite its high prevalence in the region. While sexual violence is universal, tackling it requires a deep understanding of the contextual specificities in which it arises and of the intersections of gender with other sources of oppression and marginalisation. It also calls for the recognition that intimate partner violence against women is not separate from, but linked to violence against girls, not only because both are forms of gender-based violence but because together they speak to its historical, persistent and accumulative effects.

**Methods:** In-depth intensive interviews were carried out with 35 women from Barbados and Grenada, aged 18–60 years who had experienced intimate partner violence under one of the following circumstances: during pregnancy (*n* = 15), as a woman a with disability (*n* = 8), as a woman living with HIV (*n* = 12). Interviews were digitally recorded, transcribed and thematically analyzed.

**Results:** The participants experienced multiple forms of violence within their relationships, often concurrently. Twenty-one of the women had been subject to sexual violence and of these, 19 had experienced sexual abuse as children; these experiences were viewed as interconnected and bolstered by the high level of violence-acceptance reported within communities. Women were subject to different forms of control by their partners depending upon prevailing discourses related to their circumstances (as pregnant, disabled, or HIV positive); being ‘vulnerable’ was synonymous with having one’s agency as an independent, autonomous person constrained and little external help was available.

**Conclusion:** The study identified a clear chain of sexual behaviors, each of which fuel different layers of the problem: the prevalence of early sexualization of children is associated with the prevalence of child sexual abuse; child sexual abuse is pervasive in large part, because of the normalisation and social acceptance of violence against women and girls; “cultural” normalcy, in turn, fuels attitudes which contribute to sexual violence against women and women in especially vulnerable circumstances face additional risks. Integrated policy, which tackles these as interconnected issues is called for.

## Introduction

The sexual abuse of children (CSA) is high in the Caribbean; in a survey of 15,695 students aged 10–18 years from nine countries, 47.6% of girls and 31.9% of boys reported having been subjected to sexual abuse (Halcón et al., 2003). Further, the initiation of children into sexual activity is said to occur earlier in the Caribbean than anywhere else in the world, excepting countries that practice child marriage. In surveys carried out by UNICEF, approximately 15% of children aged 11–12 years and, 35% of young people, 14–15 years old reported having had sexual experience (UNICEF, 2006; UNICEF, 2008; UNICEF, 2012). Early sexual activity is highly correlated with sexual abuse and coercion, both as a causal link and also in increasing vulnerability to further victimisation (see for instance, Barrow and Ince, 2008). In the study by Halcon et al. (2003), over half of boys and about a quarter of girls who were sexually active stated that the age of first intercourse was 10 years or younger and almost two-thirds had intercourse before the age of 13, often as a result of force or coercion. Retrospective studies with adult survivors support these findings. In Barbados, for example, 30% of female respondents had been sexually abused as children. This study, which included several countries, concluded that forced sexual initiation and early childhood abuse were not uncommon in the Caribbean (World Health Organization, 2013). Jones and Trotman Jemmott explored the perceptions, attitudes and opinions of adults in regard to CSA within six Caribbean countries and highlighted societal acceptance of violence, patriarchal values which minimise the rights of women and children and, norms which associate masculinities with domination and sexual entitlement, as key factors (2009). Violence within the home, including CSA, can contribute to the intergenerational transmission of intimate partner violence (IPV) including, sexual violence (Heise and Garcia-Moreno, 2002; Capaldi et al., 2012; Jones et al., 2014); indeed, survivors of one form of violence are more likely to be victims of other forms and, girls who have been sexually abused are more likely to experience sexual re-victimization and be a victim of IPV in adulthood (Milletich et al., 2010; Franklin and Kercher, 2012).

There is a dearth of qualitative research which explores the problem from the perspectives of Caribbean women, despite UN reports that every one of the Caribbean islands has a sexual violence rate higher than the world average (United Nation, 2007; Guedes, 2012). Also missing, are studies which examine differential experiences of sexual violence among women whose circumstances may place them at increased risk of victimisation. While sexual abuse is universal, tackling it requires attention to the local social, cultural and contextual specificities in which it arises and also, to the intersections of gender-based violence with other sources of oppression. This article makes a contribution to bridging this gap.

The study draws on the experiences of three groups of Caribbean women in “especially vulnerable circumstances” who were victims of IPV: women who were pregnant, disabled women and, women living with HIV. The study explores their experiences of sexual violence as a feature of IPV and the meanings they attribute to associations with child sexual abuse.

## Women in Especially “Vulnerable” Circumstances–a Constructivist Lens

This research uses an intersectional lens to investigate the convergence of gender, sites of “vulnerability” and difference among Caribbean women who have experienced sexual violence. Intersectionality originated as a conceptual tool to analyze the effects of race, gender and other forms of discrimination on women of color (Crenshaw, 1989) and is now widely used for the analysis of multi-layered social and institutional structures, their inter-connectedness and the ways in which they intersect with women’s gendered identities (Sumi et al., 2013; Carastathis, 2016; Hill Collins, 2019). Women in many Caribbean countries have made extraordinary strides in challenging inequalities, and human rights indicators point to the growth of women’s progress and achievements. However, despite their higher education attainment, women are less likely than men to hold positions of power, have higher levels of unemployment and are more likely to be subjected to gender-based violence, including sexual violence (UNFPA Caribbean, 2017). Furthermore, ingrained systems of patriarchy, intersecting with other structures (economic, social, and cultural traditions etc.) that together make up identities mean that many Caribbean women continue to be constrained and controlled (Jones et al., 2014). Patriarchy, viewed as symbiotic with processes of domination, provides the context in which the phrase “women in especially vulnerable circumstances” is used in this article. Un-problematized, the terms connotes victimhood rather than agency, but situated within the critical realist standpoint adopted for the study, “vulnerability” is viewed not as objective descriptor, but as a fluid state constructed at the nexus through which normative ideology (the social meanings attributed to women’s health status, in this instance as pregnant, disabled, or living with HIV) meets structural reality.

Focusing on women in especially vulnerable circumstances can unearth what might be known about sexual violence among women from diverse and marginalised backgrounds, but a critical realist perspective takes this further and insists on examining the symbolic and material structures that limit women’s freedoms. Critical realism calls also, for the recognition of human agency (Humphries, 2008). Acknowledging agentic power is important, but oftentimes analysis offers little more than a rhetorical nod in the direction of women’s empowerment. It is not enough to idealise agentic womanhood in constrained environments as heroic; we need to understand more deeply, the contexts and liminality of women’s conscious action (Hill Collins, 2000). This study thus also makes use of Einspahr (2010) structural theory of freedom which regards freedom as non-domination and calls for the dismantling of the constraining effects of patriarchy as it impacts both the material and, symbolic circumstances of women. This is crucial in tackling sexual abuse in the Caribbean since here, hegemonic versions of masculinity not only dictate what it means to be gendered, but also, what it means to be sexualised, as explored by Kempadoo and Taitt (2006) in their review of 150 journal articles, programs and “grey” literature. In respect of the entry points of analysis for this article, it also dictates what it means to be a woman who is pregnant, disabled or living with HIV.

IPV is not demographic specific and there is no typical victim. There are myriad ways in which women might be rendered vulnerable to IPV: homelessness; drug and alcohol abuse; mental ill-health; trafficking; environmental disasters; ill-health and more. There was, of course, simply no way of including all these groups in the study and through consultation with women’s organisations in the participating countries, a consensus approach was used to determine the final selection.

### Pregnancy and Intimate Partner Violence

Accurate data on IPV during pregnancy is scarce in the English-speaking Caribbean yet estimates for other Latin America and Caribbean countries are among the highest in the world (Han and Stewart, 2014) signalling this as a significant public health problem across the region (Campbell et al., 2004). IPV during pregnancy is a global problem too. In a Canadian study, 10.5% of women of the 23,766 respondents, reported being physically and/or sexually abused during pregnancy (Taillieu et al., 2015); in India, 18% of 2199 women experienced violence during their last pregnancy (Ahmed et al., 2006) and a study in Uganda revealed that of 612 Ugandan women screened in their second trimester, 27.7% had been subjected to IPV. Burch and Gallup Jr (2004) conducted research with 258 men convicted of spousal abuse and found that the severity and frequency of violence they inflicted actually doubled during their partner’s pregnancy. A meta-analysis of studies from 15 countries, suggests a prevalence rate of between one and 28%, with up to half of all victims receiving direct blows to the abdomen during pregnancy (Ellsberg et al., 2008). Though pregnancy is not of itself a source of vulnerability, it can be a catalyst for violence. (Audi et al., 2008) A Caribbean study noted that for some women, violence began when they got pregnant, while for others, men used the pregnancy situation to exert more violence and control than they had previously (Jones et al., 2017). Pregnant women are rendered vulnerable *because of* IPV, in that violence places them at extreme risk of adverse maternal and infant outcomes (Silverman et al., 2006; McMahon et al., 2011).

### Disability and Intimate Partner Violence

Both men and women with disabilities are more likely to experience violence within their personal relationships than non-disabled persons (UNAIDS/WHO, 2007) however, the cumulative effect of gender inequalities and disablism exposes women and girls to a high risk of IPV, coercion and, sexual violence (Hughes et al., 2012). A survey carried out in European Union member states reported that almost 80% of women with disabilities involved in the study had been victims of violence, and they were four times more likely to be subjected to sexual violence (la Riviére Zijdel, 2004). Breiding and Armour (2015) drew on data from the United States National Intimate Partner and Sexual Violence Survey (NISVS) (an ongoing, telephone survey of adults) and reported similar concerns. From a total of 9086 females who completed the survey in 2010, women with a disability were significantly more likely to experience rape than other women (1.7 compared to 0.4), other forms of sexual violence (4.5 compared to 1.8), physical abuse (7.1 compared to 3.3), stalking (21.0 compared to 12.2), and psychological abuse and control of sexual health (2.4 compared to 1.4) (Breiding and Armour, 2015). In the United Kingdom, research commissioned by Women’s Aid (Hague et al., 2011) involving a survey of domestic violence and, disability organisations and, interviews with 30 disabled women revealed that 50% of women with disabilities had experienced IPV compared with 25% of women without disabilities; they were twice as likely to be assaulted or raped and were likely to have to endure IPV for longer because of the lack of appropriate support. Although there are some studies on the victimization of women with disabilities in Latin America and the Caribbean, there are no reliable prevalence data. As the region has one of the highest rates of violence against women in the world, it can be assumed that Caribbean women with disabilities are disproportionately impacted by IPV because of limited access to services and the position of power and control abusers may have over them. Disabled women are also likely to be more dependent on their abuser for care and support than other groups of women and have reduced access to economic autonomy; these factors exacerbate isolation and vulnerability.

### Living With HIV and Intimate Partner Violence

Violence against women and HIV are conjoined public health problems with profound implications for health, wellbeing and social development. The Caribbean has the highest incidence of HIV-AIDS outside of Africa and it is estimated that 53% of persons living with HIV in the region are women (Allen, 2011). Although women are biologically more susceptible to contracting HIV infection, this does not fully explain how and why HIV has become so heavily feminized. Global studies reveal that this is primarily due to gender inequalities, sexual cultures, violence against women and girls and women’s lack of control over their sexual rights. Forced sex and rape, place women and girls at particular risk, as these types of sex cause injuries in the vaginal and anal tissue, allowing the virus to be more easily introduced into the body (Luciano, 2013). HIV transmission in the Caribbean is increased by complex sexual cultures which include: the early sexual initiation of girls; sexual exploitation; forced, non-consensual sex; norms about male sexual entitlement; multiple partnering and, sexual-economic exchange relations. This construction of complex sexual cultures is supported by qualitative research with sex workers (Kempadoo, 2004), with adolescent girls 16–18 years (Barrow 2008) and also, through a review of literature conducted by Bombereau and Allen (2008). At the ideological level, HIV and AIDS continue to symbolize deviancy and contagion and, in the Caribbean, women living with HIV face stigmatization, social alienation and victim-blaming. These factors force women into silence and often cause them to have to mask violence within relationships. Violence against women therefore has both a causal and consequential relationship with HIV. There is a high level of awareness of these intersections between HIV and IPV among Caribbean governments, and international organizations (UN Women, PAHO/WHO, 2007) have initiated programs designed to catalyse integrated responses, nevertheless, policy and programming tend to be fragmented and the mechanisms for effective implementation are often missing.

### The Present Study

The empirical data presented in this article was collected in 2016 as part of a larger research project on the experiences of IPV among women in Barbados and Grenada. The study utilized a cross-sectional qualitative design–data collected at one time-point through guided reflective interviews. The objectives of the study were derived from a preliminary literature review and sought to 1) explore the ways in which women from especially “vulnerable” groups who experience IPV may be exposed to heightened risks as a consequence of their partners’ exploitation their situations and, 2) to see what commonalities or differences exist between the situations of the women.

## Methods and Materials

Participants were recruited through professional networks, agencies and snowballing. Interviews took between one-two hours and were digitally recorded. Interview prompts were designed to facilitate extended narratives, to elicit information about patterns of abuse and sexual violence experienced and to evoke memories of childhood. These prompts were: 1) How do you define domestic violence? 2) Do you face particular risks of violence because of your health or situation? 3) What do you think the reasons are for the increase in risk? 4) How does being pregnant/disabled/living with HIV affect you in getting support in dealing with or escaping a violent relationship? 5) What helps you to cope?

### Participants

Thirty-five women participated in the study: 15 of the women were pregnant, eight were disabled and 12 were living with HIV (four of these women were also pregnant). The ages of the participants at the time of the interviews ranged from 18 to 60 years. Twenty of the women were single, separated or divorced and 15 women were married or in a long-term relationship. Many women were able to participate in the study only because they had been able to escape the relationship in which they were abused and this accounts for the large number of single women in the study. Twenty-eight women had children and 13 women had four children or more. Thirty-one of the participants described themselves as poor or low income (a limitation of the sampling approach, which recruited participants through agencies that primarily provide services to poorer women), three women were of average income and one woman reported above average means.

### Procedure

This study used convenience, purposive, non-probability sampling techniques in order to identify participants whose circumstances were appropriate for the research objective. A data base of agencies and government departments providing services to the relevant groups of women in the two countries was created. Agencies that agreed to support the study distributed information about the research to their service users. Women notified the agency of their wish to be included in the study and initial contacts by email were followed up with confirmatory telephone calls. To ensure participant safety, no direct contact was made with the women and interviews were arranged through the organisation by three research assistants recruited and trained in conducting IPV research. This approach was necessary given the sensitivity of the issue explored and the potential risks to women of more representative and open recruitment methods. The recordings were transcribed verbatim and subject to iterative, in-depth, thematic analysis (Braun and Clarke, 2013). This involved three stages: first, multiple, purposeful readings of the transcripts and repeated listening to the recordings without coding to gain an overall impression of meanings of expression in the context in which they were used; second, detailed coding to identify significant themes on sexual violence as a feature of IPV among the three groups of women (pregnant women, women with disabilities and, women living with HIV) and third, the merging of ideas to produce thematic maps (Braun and Clarke 2013) and to check for patterns across groups of participants and across both countries. Once the themes were identified, quotes were selected to best illustrate the findings.

### Ethical Statement

Ethical approval for the study was granted by the ethics committee at the United Kingdom university leading the research. The participants’ identities were anonymised to minimise risk to them and counselling was made available in the event of re-traumatisation. Participants were provided with an information sheet detailing the nature of the study and informed that they could withdraw at any stage of the process. Informed consent was obtained on this basis. Data collection and data management were subject to stringent ethical protocols to safeguard women’s privacy rights and all transcripts and digital recordings were secured through password protection.

## Results

The results are organised around the three situational groupings reflected in the study: 1) Being pregnant, 2) Women with disabilities and 3) Women living with HIV. The three situational groupings, though initially predetermined based on the research objectives, were considered important to retain because these categories contextualised and differentiated women’s experiences in ways that mattered to them. In seeking to preserve difference while at the same time exploring commonality, within these groupings, three overarching cross-cutting themes are discussed: Intimate Partner Violence, Sexual Violence and Freedom. The first two of these are a product of the analysis. IPV is used to refer collectively to the many forms of violence that were inflicted within the context of the women’s intimate partner relationships; sexual violence refers specifically to violence of a sexual nature. Of the 35 women in the study who had experienced IPV, 21 reported that these experiences had included sexual violence and 19 referred to childhood experiences of sexual abuse although in two cases, the women did not view this as abuse (these two women had been raped as children). Other terms used within the analysis include physical violence, which refers to the use of physical force and, psychological violence which includes verbal and emotional abuse and threats of harm. It is important to stress that the revelations of child sexual abuse (CSA) were an emergent phenomena from this broader study of IPV and that in the discussion of results on CSA, only data from the 19 participants who reported this is explored.

**FIGURE 1:**
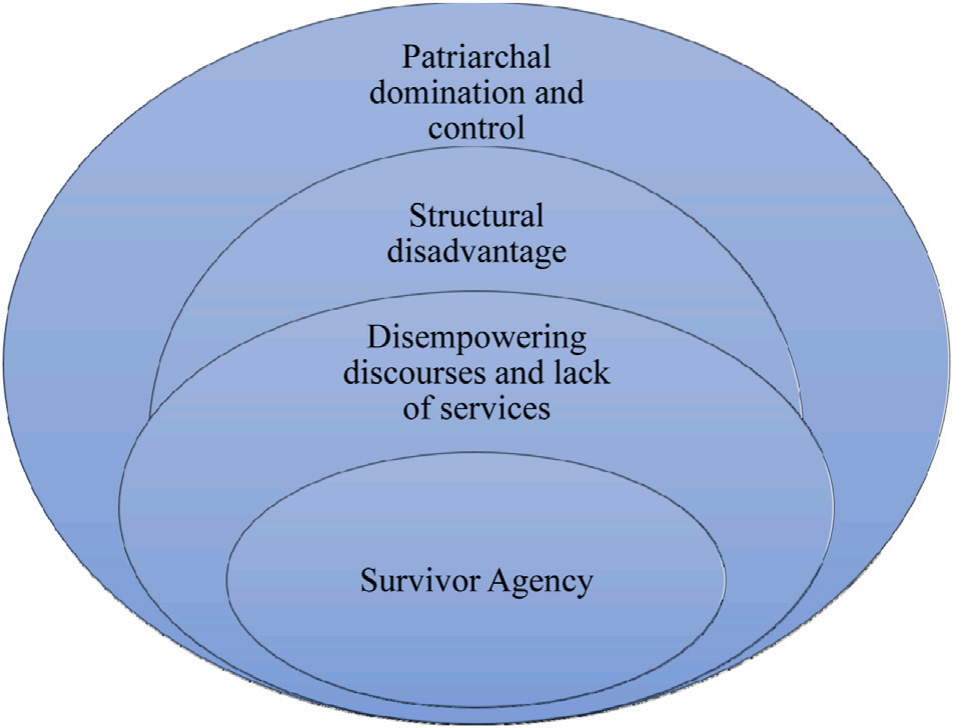
Liminal survivor agency.

It is important to acknowledge that these distinctions are a theoretical contrivance, which though seeking to provide a comparative picture of women’s experiences, runs the risk of underplaying the embeddedness of different types of violence, one within the other. In mitigating this risk it is important to stress therefore, that all the participants experienced multiple forms of violence within their relationships, often concurrently. The women did not describe these as separate forms of violence but rather, as violence with many “heads and shapes”. So, for example, physical violence exerted in order to carry out sexual violence was most likely accompanied by verbal abuse. These were not experienced as three types of violence but as single violent acts containing these different and overlapping elements. Verbal battering by itself seems to have been an ever-present feature of most women’s experiences but when linked to other violent actions, it served specific functions: a precursor to an assault, part of the build-up; a form of ‘justification’ for the perpetrator’s benefit; or, fuelling the severity of violence (e.g., reminding the victim and perpetrator of the woman’s transgressions). The third theme ‘freedom’ was theoretically derived to explore the concept of freedom as non-domination or being free from the constraining effects of patriarchy (Einspahr, 2010). Freedom meant different things for different women depending upon their situations but for all women, was centered around how to escape the violence. This in turn meant how women tried to free themselves from patriarchal domination, to challenge the structural disadvantages they faced, and to confront disempowering discourses and the lack of services. In the absence of being able to escape, it also meant the strategies women used when forced to adapt to living within these constraints which included making incremental improvements to their circumstances (liminal agency). Liminal agency as described in this study, can therefore be understood as survivors enacting agency in ways which improved their day to day lives but bounded *within* external structural barriers, were unable to disrupt them. This is represented in Figure 1.

In addition to the separate groups of women and the common themes across them, two other themes relating explicitly to sexual violence are explored: sexual abuse as lifespan trauma and the normalisation of sexual abuse. These themes dominated the narratives of 19 of the 35 women and relate to childhood experiences of sexual violence.

### Being Pregnant

#### Intimate Partner Violence

Motherhood was an important role for the majority of the women. Twenty-eight of the 35 participants had children and 13 women had four children or more. Half of the mothers (*n* = 14) had been subjected to IPV during their pregnancies and 12 women reported that the violence either started or increased during pregnancy and then persisted throughout the relationship. Emma, a 39 year old married woman with two children, experienced abuse from the third month of her second pregnancy:

*“When I get home from work tired, my husband would start cursing and abusing me. I cry a lot and sometimes I think of committing suicide. I blame myself a lot. I think I should have been able to see that it was not love. When someone loves you, they don’t hit you. I was not happy and I became very timid”*.

Most of the abuse reported was physical and emotional:

*“He dragged me in the grass and punched me. My baby came at seven months, because of the constant verbal abuse and one incident of physical abuse.”* (Vera, 49 years)

Women’s abdomens and unborn babies were often targeted to inflict maximum harm:

*“He grab me by my throat ….and push me up against the wall … then he cuff [punched] me in my stomach. He said you don’t deserve no child and I am killing you tonight”.* (Joan).

*“You cannot fight back because you have to study like, to hide my belly to prevent it being hit. It is hard to fight back; I would study to run but I could not get nowhere because I had a belly”.* (Betty)

*“One day he gave me a blow in my temple and I fell to the ground and my cervix tore. I began to bleed and had to be rushed to the hospital. My cervix had to be stitched up to hold the baby for the balance of the pregnancy.” *(Peace).

#### Sexual Violence

Sexual violence was also commonly reported among pregnant women. Two women had become pregnant as teenagers as a result of rape while other women said they faced sexual assaults right through and immediately after their pregnancies. Jenny recounted:

*“…he wanted sex. I told him the baby is too young and I can’t go through with that. He took a knife and he cut off my clothing.”*

*“Once he [describes violent vaginal sexual assault] and tell me that he will rip the child from inside me.”* (Maureen)

#### Freedom (Constraints to Escaping Violence)

Mothers were reluctant to leave violent partners because it was important to them that their children should not grow up without a father. This was especially the case for women whose pregnancy had seemed to be the catalyst for violence. These women convinced themselves that once the child was born the violence would stop; they were also encouraged by the family to remain in the relationship for the “sake of the baby”. Participants who had endured abuse over time recalled feeling it was a “phase” that would pass but were often able to pinpoint the time when they realised it would “never stop”. This was most often when they became aware that their children were being directly affected. The participants believed it was important for women to get out of abusive relationships as soon as the abuse starts, regardless of whether one was pregnant or not and yet they also recognised that physical and social constraints made this extremely difficult especially when there were other children. In instances where women left their partners, they often felt compelled to return because there was nowhere to get help and without a partner, they were victim to social disapproval, as Vera explains:

*“People are judgmental so it will be hard for persons to get help when pregnant”*.

Andrea, a 39 year old woman living with HIV, was abused during her three pregnancies. She said she that she could not go anywhere because she was “big pregnant” and all she wanted to do was to protect her baby. She gained temporary respite from the beatings when the neighbors hid her, but she always had to return.

### Women With Disabilities

#### Intimate Partner Violence

The experiences of women with disabilities revealed that social and structural isolation compounded the violence they were exposed to. Intimate partner violence often entailed the exploitation of specific impairments as a means of inflicting psychological harm. These women did not feel limited by their impairments but their partners would make use of their impairments to constrain them and *this* was limiting. Aya, was blind and when her husband wanted to punish her, he would move all of her markers of space and place in the home so that she would be lost, stumble or require his assistance. When Cathy, aged forty-one and a mother of five children had a stroke, her husband became abusive. In addition to physical and sexual abuse, he told people she had had a “nervous breakdown” and was mentally unstable. He was so plausible that friends began to shun her and even the courts believed him.

*“Many times, he forced me to have sex. When I told him that I would expose him and tell everyone that he is abusing me he told me that he would tell everyone I am mad. This was the beginning of the campaign to make everyone believe I was mad since the stroke”*.

Michelle had been badly burned in a fire and was visibly scarred; she was left with physical impairments. A survivor of physical and sexual violence, she had actually been burned saving the life of another female victim of violence. Despite the IPV she was experiencing in her current relationship, this act was recalled with pride and seemed symbolic of her resilience–she had no doubt that she would leave as soon as she “saved a little money”.

*“A man wanted to kill her. I saved her, I put her on my back and brought her out. I was 22 years when this happened…. They were fighting and the man came home and light the fire in the night. I was in the newspaper when I got burn up in the fire.”*

#### Sexual Violence

Women with disabilities seemed to be at particular risk of sexual violence, especially if their partners had a role in their personal care. This violence was sometimes “opportunistic”, inflicted when helping the woman with her personal care; women reported that it felt like their bodies did not belong to them but were simply there for the sexual gratification of their partners. Other women said their partners expected them to show gratitude, including sexual gratitude, in return for being provided with somewhere to live and food. Aya, explained that because she was completely reliant upon her husband and had nowhere else to go, she believed she had no choice but to endure the abuse. She also described a longstanding pattern of sexual exploitation both inside and outside the home.

*“I’ve had of people pushing themselves at me sexually because I was dependent on them for money”*.

Cathy, like some other women with disabilities, did not feel she could ever deny her husband sex although he made her feel that her disability rendered her sexually unattractive. Paradoxically, he continually accused her of “looking for sex” whenever she ventured out of the home without him. Cathy was routinely sexually violated by her husband as a means of surveillance.

*“Every time I go out, he would want to know where I went and [describes being sexually assaulted] to see if I was having sex with someone else. I could not go anywhere without him thinking I was seeing someone”*.

#### Freedom (Constraints to Escaping Violence)

Women with disabilities, like the other women in the study spoke of having to depend on their abuser for economic and other forms of assistance. Material dependency was compounded for some disabled women by the psychological dependency borne out of chronic abuse and the lack of services to facilitate their independence. Reduced personal autonomy resulted in high levels of control and domination; this created both physical and psychological barriers to leaving.

*“…how can I tell anyone about him when I need him to help me get up, to bathe, to go to the toilet; how can I leave if I cannot live on my own?”* (Sasha).

Peace was locked in the house while her partner went to work. There were times when he forgot to take the keys but as she was not independently mobile, she was unable to leave and in any event was so fearful of what he would do upon finding her that she did not consider this a viable option. Having grown up with an acute awareness of IPV from witnessing it in her own family, Michelle struggled to escape it. As a disabled woman, “things only got worse”. She was finally able to leave when someone called the police when she was being held captive.

*“He would tie me up to the bed and have sex with me when I was tied up. All the women that he has he beats them and tie them up and rapes them”*.

Tracy, a 20 year old single woman with a physical disability had been totally reliant on her father since being an infant. She was not permitted to use a wheelchair and in the absence of external help, had no means of escaping the sexual violence he subjected her to.

### Living With HIV

#### Intimate Partner Violence

As with other participants, women living with HIV suffered all forms of IPV, in addition however, they also lived with the constant fear that their partner would disclose their HIV status. In a context in which HIV-AIDS stigma and discrimination can adversely affect all aspects of social life (employment, friendships, leisure activities) not only for the woman, but for her children, this was a powerful psychological weapon which inflicted a great deal of harm and fueled feelings of low self-worth. Agnes, a 56 year-old woman living with HIV believed she acquired the virus from her husband as he had a lot of sexual partners (although, to her knowledge, he had not been tested). She said that he continually mocked her and regularly threatened to tell their friends.

*“…he started insulting me in public. Like, he would make a big fuss about not using the same glass or fork... Everybody would hear him and you could see what they were thinking. I asked him, please don’t say that … , they will think I have some terrible disease. And he would say well you DO have a terrible disease”*.

#### Sexual Violence

For some women, sexual violence had been the pathway through which they had acquired the virus. Sula, a married woman aged 59 years had been living with HIV for many years. She said she had first been abused at the age of 9 years and this led to a pattern of engaging in high risk sexual behavior during adolescence and as a young adult. Although she had been a child, Sula did not view her early sexual encounters as coercive. She referred to being raped as a young girl as “having sex” and described herself as “simply going along with men who would come on to me, you know”.

Annie did not know she was HIV positive until she attended the maternity clinic when she was pregnant. Her husband had been working away and during this time, she had a sexual relationship with someone she met at college. She was devastated by her diagnosis and believed that she deserved to suffer for her infidelity.

*“My husband never looked at me the same again. Although I think he always loved me. But he could never see me the same again. Then he started having sex with me again but only anally. Very hard. Very rough. Like an animal. Like a punishment”.* (Annie).

Andrea, a 37 year old mother of three children, had experienced sexual abuse as a child and as an adolescent. She also lost a child to abuse; her two-year old daughter died after being molested by an uncle. In her last relationship, she was “pimped” by her boyfriend; he refused to work and sent her out prostituting to pay the bills and buy food and drugs, she was beaten often.

*“I sold my body to pay for drugs, I was assaulted by many customers; it was a very bad time”*.

Andrea acquired HIV from one of the male customers.

#### Freedom (Constraints to Escaping Violence)

Women living with HIV often experienced high levels of stigma and alienation from families and their communities. This compounded IPV in specific ways and as their partners also exploited their circumstances, increased their vulnerability to ongoing abuse. Some women chose to put up with the abuse, rather than facing the social trauma of entering into a new relationship and having to disclose their HIV status. Several participants said that their partners threatened to disclose their status if they left and they considered that living with IPV was better than facing the unknown consequences of HIV disclosure. Some women had lost jobs because someone had disclosed their status, and the fear of not being able to provide for themselves and their children meant that survival required they develop the personal resilience to remain in the relationship. For these women, the home, though a place of violence, paradoxically provided a ‘safety-net’ against the risk of being homeless and unemployed as a woman living with HIV who has children. For Andrea, it was not fear of others learning of her HIV status that forced her to stay in an abusive relationship, but her addiction to drugs. She had started using drugs as a way of dealing with the abuse and death of her two-old daughter:

*“I smoked and smoked and smoked and smoked and blocked out everything’, all of the pain, the abuse”*.

Andrea’s dependency upon crack cocaine was sustained by the fact that her partner was also using drugs. Andrea was one of the few women in the study who had been able to access appropriate support. She was sent to a drug rehabilitation facility (where she was living at the time of the interview) and was working to create an independent abuse-free life for herself.

*“Right now, I am a real survivor for real. Because after 16 years of abuse in every shape and form I am currently doing two courses, one in jewellery making and the other one in auxiliary nursing”*.

### Sexual Abuse as Lifespan Trauma

This section of the article draws only on the data of the 19 women who revealed that as well as facing IPV as adults, they had been subjected to child sexual abuse.

As in Andrea’s circumstances (described in the previous section), women who reported sexual violence as adults, in almost all cases, also referred to historic child sexual abuse and for some, sexual violence had continued across the span of their lives. Cynthia, a 39* *year old married, but separated woman was sexually abused by an adolescent boy who was a close friend of the family when she was 11. Debbie, a twenty-six-year-old single mother with two children also said that her abusive experiences began in childhood; she was abused by her uncle when she was 10* *years old. As with Cynthia and Debbie, several women questioned whether their later experiences of sexual violence had been in part “scripted” because of the failure of adults to believe them when they were children and the ways in which the abuse was “brushed over”. This was the case for Sami, a 38* *year old single mother of eight. She recalled being molested by her uncle when she was about six and then she was raped (vaginally and anally) at around 10* *years of age by her sister’s boyfriend. When she was 14, Sami ran away with 20* *year-old a man to whom she became pregnant. Having experienced sexual molestation, rape and sexual exploitation at different stages during her childhood, she had gone on to experience sexual violence in every one of her adult relationships. Sami believed that her early sexualisation and abuse had led to a lack of self-worth and a view of herself as having no value outside of being available to service men’s sexual desires; she wondered whether this contributed to the poor relationship choices she said she had made.

Some women recalled witnessing their mothers being sexually abused when they were children and later, as adults, being subjected to abuse in front of their own children.

*“He started abusing me, physically he would beat and hit. Then sexually where he would come home at night and rape me in front of our daughter”*.

Interviewer: How old was she?

*“She was three and she would be up. It was like seeing a cycle of my mother when I was a child and that really hurt me”*.

### Normalization of Sexual Abuse

Sami and the other women who had experienced abuse in childhood questioned whether the environments in which they had grown up in had laid the groundwork for their victimisation.

Debbie, sexually abused by an uncle, later found out her mother had also been abused by an uncle.

*“It was a generational thing because his father did it to my mother and he continued on that trait”*.

Several of the women described living in households in which male sexual entitlement was an accepted social norm. In the absence of oppositional voices, the oftentimes complicity of families and communities and, the failure of child protective services to recognize and act on abuse, these norms became embedded within everyday life. Tracy, a 20* *year old disabled woman said she was in primary school when she was first sexually abused by her father. With a severe mobility impairment, Tracy was carried everywhere by her father; she did not have a wheelchair. From her being very young, her father assumed responsibility for her personal care needs. After the first incident of sexual abuse, this became a part of the daily routine of “care” he provided. Tracy said she loved her father and did not see a problem with what he was doing. She also revealed that she had become pregnant with her father’s child and had been forced to have an abortion. Her response to the interviewer on how she felt about this was “I am only grateful. Can you imagine?” Physical and economic dependency intersecting with patriarchal domination and an assumption that Tracy has no rights to her own body or sexuality, had created a life of which, she could not imagine existing outside. When a family member suggested Tracy should consider emigrating so that she could go to college and get support to live independently. She retorted: “Home is fine for me”.

Sula too, had grown up thinking that it was normal for men to have sex with young girls.

*“…we have a lot of grown men having sex with young girls. It’s normal. I don’t know any young girls who did not have sex with men. It was normal”*.

Her initiation into sexual submissiveness was to have a profound effect on her sexual behavior as an adult, leading to her acquiring HIV.

Most women who had witnessed abuse as children or who had been victimized themselves, recognized the behavior as abusive but in the face of societal acceptance, did not think they had the power to do anything about it.

*“I realized it was abuse as soon as it started, but because I grew up in an abusive home, I thought that this is how it should be. My mom went through it so I thought that I had to do the same”.* (Emma)

Some women recalled being oppositional and defiant and several reported abuse to others even though this sometimes resulted in them being disbelieved or being blamed.

*“… I remember plainly my first words were “mommy uncle M … interfere with me”. I could remember being called a [offensive ] and a [offensive ] and being told that I want killing, at two years old. So, if you at two years hear that, ok, things die down for a while then he started back. If you tell somebody in the beginning and nobody believed you who will you tell again?”*

Aya lost her sight at the age of 11 and soon after, her father started sexually abusing her; she refused to keep quiet about it: “I spoke to my grandmother, I spoke to my mother and, I spoke to my teacher”. After two years, the abuse stopped, although Aya was made to feel responsible for what had happened.

*“I don’t know how to explain how it affected them because they never even stood up for me. All of them were against me”*.

The normalization of sexual abuse meant that there were rarely any negative consequences for the men who perpetrated it. Michelle had become pregnant as a result of coercive sex when she was 15 and railed against the fact that while her abuser faced no consequences, she was ostracized and given no support.

“…*you know as a teenage mother … you are so stigmatized and discriminated against and people tend to judge you when they do not know the circumstances of how you became pregnant, so I faced all of that and my life started going downhill from then”.*

Michelle was also angry that she had been ignored when she first reported CSA as a young girl and believed that had help been available, she may have been able to avoid the series of abusive relationships she later encountered. She was particularly distressed that at 15, she had been expelled from school because she was pregnant and was not able to complete her education. She had nevertheless demonstrated considerable resilience and now, as a mother, she was determined that though her children had been exposed to violence, she would do all she could to make sure they would not be at risk of violence in their own lives.

## Discussion

This article has explored the intersections of patriarchal domination, structural disadvantage and interpersonal violence among three groups of women in especially vulnerable circumstances in Barbados and Grenada. The experience of violence associated with being pregnant, disabled or, living with HIV was unique to each individual woman however, the axes of differentiation that structured the women’s lives were connected to each other because of systemic inequalities. In applying an intersectional lens, a common theme to emerge from the findings was that women’s material circumstances and the extent to which they were subject to control and constraint was related to the discourses which surrounded their social status. Weaponizing of difference and disadvantage was a central feature of many women’s experiences and perpetrators of violence often exploited a woman’s impairment or situation in order to humiliate and exert control. Being “especially vulnerable” was synonymous with having one’s agency as an independent, autonomous person constrained and suppressed. These constraints were impacted by physical and social restrictions and through the interplay of patriarchal power with institutional failings. Two agentic factors emerged from the findings: women’s economic dependency and, the psychological dependency generated by ideologies of female submission. At the time of the interviews, some of the participants had been able to generate a modest income through crafts or selling, however most had been financially reliant upon their partners when they experienced violence and this severely limited agency and opportunity for escape. All of the women in the study bemoaned the lack of services to support victims of violence in acquiring the means to live independently. Women needed help to live; this, they considered, was the best way of helping them to leave.

### Patriarchy and Constraints on Leaving Violent Relationships

In identifying the plurality of power relations in the experiences of women in especially vulnerable circumstances, it was clear that cycles of continuous and coterminous oppression and violence are very difficult to exit without external support. Implicit in patriarchal role expectations that were a feature of the lives of the participants, was the requirement for women to adopt subject positions that embody acquiescence and sexual availability. Normative discourses around being pregnant, disabled or living with HIV and the symbolic meanings they generated, created additional social barriers to leaving violent relationships. So, for example, expectations concerning maternal roles were found to be predicated on essentializing views of motherhood (Jeremiah, 2006). If a woman was pregnant and the father of her baby had not abandoned her, then she simply could not leave, regardless of abuse or any other threats she faced; the role is considered immutable and so are its social requirements (Jeremiah, 2006). The experiences of women with disabilities, revealed that disability is often openly parodied in ways that served to diminish them. These women described being the target of fun, pity, and discrimination not only in their personal lives, but in their interactions with public agencies (the police, the courts, welfare officials). This aligns with other research on the topic (e.g., Brownridge, 2006; Breiding and Armour, 2015). Prevailing paternalistic attitudes undermined disabled women’s rights to autonomy; treated as childlike and lacking intellectual capacity, their accounts of violence were often regarded as non-credible and they were systematically denied access to the means of self-determination. Disabled women could not leave violent relationships because they were assumed not to be able to cope on their own and there were few services to enable this, even in the face of the violence they suffered. Women living with HIV face extremes of stigma and discrimination spanning everyday microaggressions that communicate social contempt, right through to overt discrimination and violence (Hale and Vazquez, 2011; Luciano, 2013). That hegemonic perceptions associate HIV with deviance, contagion and sanction was evident in the women’s accounts. Even though the Caribbean has some excellent public education programmes aimed at tackling HIV discrimination, women believed that disclosure of their status would lead to them being shunned and targeted and would generate huge problems for their children. Women living with HIV cannot leave violent relationships because the fear of the consequences is simply too great.

### The “Ties That Bind Us”–Early Sexualization, Child Sexual Abuse, Cultural Normalcy and Sexual Violence Against Women

In the present study, women’s experiences of IPV were exacerbated because of a high level of tolerance for violence against women and girls within their families and communities and, the acceptance of early sexualization. This link between violence victimization in childhood and violence acceptance is confirmed through research on attitudes and exposure to abuse among 1400 children in Barbados and Grenada (Debowska et al., 2018) and studies on the prevalence of interpersonal violence (Le Franc et al., 2008). Furthermore, Barrow and Ince (2008) demonstrate that sexual exploitation is strongly associated with children becoming sexually active at a young age. Other studies have also reported the association between social acceptance, cultures of normalcy and child sexual exploitation in Caribbean settings (e.g., Kempadoo and Taitt, 2006; Barrow 2008). In the present study, 17 of the 19 participants who recalled sexual victimization as children suggested that these earlier violations, set within a context in which sexual relations between children and adults sometimes were normalized, may have contributed to the conditions for later IPV they faced (two participants did not make this link). Described by one woman as “the ties that bind us”, this author contends that the interrelationship between violence against children and violence against women is a key finding, one which can inform policy and the development of more cohesive services. This is supported by other studies (see, for example, Namy et al., 2017).

Schröttle and Glammeier (2013) suggest that the risk of IPV is increased two/three times when women have experienced violence in childhood since these early experiences can lead to low self-esteem and undermine the ability to set boundaries in relationships. The relationship choices women in especially vulnerable circumstances make in such circumstances cannot be deemed wholly agentic. This is because 1) these choices are predicated *on* and situated *within* wider ideological norms which promote patriarchal values of dominance, male sexual entitlement and female acquiescence (Harris et al., 2005; Ahrens et al., 2010) and 2) the women making these choices are subject to material, psychological and structural constraints arising out of the intersections of gender and social status over which they have little control (Heise et al., 1998; Hale and Vazquez, 2011). Combined, these processes fuel the prevalence and persistence of violence against women and limit agency. The current study has focused on women who are pregnant, disabled or, living with HIV, however the concept of “ties that bind us” is an important one for all women. Greater recognition of sexual abuse as lifespan trauma and sustained integrated action on violence prevention for children *and* women is therefore essential.

## Strengths and Limitations

The strengths of the current study include the foregrounding of differential experience of sexual violence within a unifying intersectional framework which includes attention to patriarchy, agency and social circumstances. This has revealed unique insights into the specificities of what is a universal problem. Limitations of the study lie in the sampling methods used, for example, only participants who had experienced IPV were included and only women who were in contact with a support agency were included. This means that the voices of women unable or unwilling to access services or, who may be prevented from accessing services have not been included in this study; women who may be at even greater risk of violence than those in contact with NGOs. Research is also needed among women who are pregnant, disabled and living with HIV and who are *not* subject to IPV to determine the ways in which the intersectional framework can generate insights into lives not impacted by violence.

## Data Availability

The datasets presented in this article are not readily available because this dataset includes transcripts of qualitative interviews with women, several of whom were in violent relationships at the time of the research; given the small size of the countries in which the study was conducted, there is a risk that some of the women may be identifiable and this could jeopardize their safety. Requests to access the datasets should be directed to a.d.jones@hud.ac.uk.

## References

[B1] Ahmed S. Koenig M. A. Stephenson R. (2006). Effects of Domestic Violence on Perinatal and Early-Childhood Mortality: Evidence from North India. Am. J. Public Health 96 (8), 1423–1428. 10.2105/ajph.2005.066316 16809594 PMC1522123

[B2] Ahrens C. E. Rios-Mandel L. C. Isas L. del Carmen Lopez M. (2010). Talking about Interpersonal Violence: Cultural Influences on Latinas’ Identification and Disclosure of Sexual Assault and Intimate Partner Violence. Psychol. Trauma Theor. Res. Pract. Pol. 2 (4), 284–295. 10.1037/a0018605

[B4] Allen C. F. (2011). Intersections between HIV/AIDS and Violence against Women: Research to Develop Pilot Projects in Barbados and Dominica. J. East. Caribbean Stud. 36 (4), 39–59. Retrieved from http://search.proquest.com/docview/1040723971?accountid=11526.

[B5] Audi C. A. F. Segall-Corrêa A. M. Santiago S. M. Andrade M. d. G. G. Pèrez-Escamila R. (2008). Violência doméstica na gravidez: prevalência e fatores associados. Rev. Saúde Pública 42 (5), 877–885. 10.1590/s0034-89102008000500013 18695785

[B6] Barrow C. Ince M. (2008). Working Papers in Early Childhood Development #47: Early Childhood in the Caribbean. The Hague, Netherlands: Bernard van Leer Foundation.

[B7] Barrow C. (2008). Sexual Identity, HIV, and Adolescent Girls in Barbados. Soc. Econ. Stud. 57 (2), 7–26. Retrieved April 21, 2021, from http://www.jstor.org/stable/27866549.

[B8] Bombereau G. Allen C. (2008). Social and Cultural Factors Driving the HIV Epidemic in the Caribbean Literature Review. St. Augustine, Trinidad and Tobago: Caribbean Health Research Council.

[B9] Braun V. Clarke V. (2013). Successful Qualitative Research: A Practical Guide for Beginners. London: SAGE Publications Ltd.

[B10] Breiding M. J. Armour B. S. (2015). The Association between Disability and Intimate Partner Violence in the United States. Ann. Epidemiol. 25 (6), 455–457. Available at www.annalsofepidemiology.org/article/S1047-2797(15)00127-1/fulltext. 10.1016/j.annepidem.2015.03.017 25976023 PMC4692458

[B11] Brownridge D. A. (2006). Partner Violence against Women with Disabilities. Violence Against Women 12 (9), 805–822. Available at http://journals.sagepub.com/doi/abs/10.1177/1077801206292681. 10.1177/1077801206292681 16905674

[B12] Burch R. L. Gallup Jr. G. G. (2004). Pregnancy as a Stimulus for Domestic Violence. J. Fam. Violence 19 (4), 243–247. 10.1023/b:jofv.0000032634.40840.48

[B13] Campbell J. García-Moreno C. Sharps P. (2004). Abuse during Pregnancy in Industrialized and Developing Countries. Violence Against Women 10 (7), 770–789. 10.1177/1077801204265551

[B14] Capaldi D. M. Knoble N. B. Shortt J. W. Kim H. K. (2012). A Systematic Review of Risk Factors for Intimate Partner Violence. Partner Abuse 3 (2), 231–280. 10.1891/1946-6560.3.2.231 22754606 PMC3384540

[B15] Carastathis A. (2016). Intersectionality: Origins, Contestations, Horizo*ns*. Lincoln: University of Nebraska Press. 10.2307/j.ctt1fzhfz8

[B16] Crenshaw K. (1989). Demarginalizing the Intersection of Race and Sex: A Black Feminist Critique of Antidiscrimination Doctrine, Feminist Theory and Antiracist Politics. University of Chicago Legal Forum Article 8. Available at: https://chicagounbound.uchicago.edu/uclf/vol1989/iss1/8.

[B17] Debowska A. Boduszek D. Sherretts N. Willmott D. Jones A. D. (2018). Profiles and Behavioral Consequences of Child Abuse Among Adolescent Girls and Boys from Barbados and Grenada. Child. Abuse Neglect 79, 245–258. 10.1016/j.chiabu.2018.02.018 29486347

[B18] Einspahr J. (2010). Structural Domination and Structural Freedom: a Feminist Perspective. Fem. Rev. 94 (1), 1–19. 10.1057/fr.2009.40

[B19] Ellsberg M. Jansen H. A. Heise L. Watts C. H. Garcia-Moreno C. (2008). Intimate Partner Violence and Women’s Physical and Mental Health in the WHO Multi-Country Study on Women’s Health and Domestic Violence: an Observational Study. The Lancet 371 (9619), 1165–1172. 10.1016/s0140-6736(08)60522-x 18395577

[B20] Franklin C. A. Kercher G. A. (2012). The Intergenerational Transmission of Intimate Partner Violence: Differentiating Correlates in a Random Community Sample. J. Fam. Viol. 27, 187–199. 10.1007/s10896-012-9419-3

[B21] Guedes A. (2012). Violence against Women in Latin America and the Caribbean: A Comparative Analysis of Population‐based Data from 12 Countries. Published 2011. http://www.igwg.org/igwg_media/guedes‐gbv‐lac.pdf.

[B22] Hague G. Thiara R. Mullender A. (2011). Disabled Women, Domestic Violence and Social Care: The Risk of Isolation, Vulnerability and Neglect. Br. J. Soc. Work 41 (1), 148–165. Retrieved April 21, 2021, from http://www.jstor.org/stable/43772532. 10.1093/bjsw/bcq057

[B23] Halcón L. Blum R. W. Beuhring T. Pate E. Campbell-Forrester S. Venema A. (2003). Adolescent Health in the Caribbean: A Regional Portrait. Am. J. Public Health 93 (11), 1851–1857. 10.2105/AJPH.93.11.1851. 10.2105/AJPH.93.11.1851 14600052 PMC1448062

[B24] Hale F. Vasquez M. (2011). Violence against Women Living with HIV/AIDS A Background Paper. Washington, DC: Development Connections (DVCN). http://salamandertrust.net/wpcontent/uploads/2012/12/VAPositiveWomenBkgrdPaperMarch2011.pdf.

[B25] Han A. Stewart D. E. (2014). Maternal and Fetal Outcomes of Intimate Partner Violence Associated with Pregnancy in the Latin American and Caribbean Region. Int. J. Gynecol. Obstet. 124 (1), 6–11. 10.1016/j.ijgo.2013.06.037 24182684

[B26] Harris R. J. Firestone J. M. Vega W. A. (2005). The Interaction of Country of Origin, Acculturation, and Gender Role Ideology on Wife Abuse*. Social Sci. Q. 86 (2), 463–483. 10.1111/j.0038-4941.2005.00313.x

[B27] Heise L. Garcia-Moreno C. (2002). “Violence by Intimate Partners,” in World Report on Violence and Health. Editors Krug E. Dahlberg L. L. Mercy J. A. Zwi A. B. Lozano R. (Geneva, Switzerland: World Health Organization), 87–121.

[B28] Heise L. Ellsberg M. Gottemoeller M. (1999). Ending Violence against Women. Popul. Rep. L. (11), 1–43. Baltimore: John Hopkins University School of Public Health, Population Information Program.11056940

[B29] Hill Collins P. (2000). Black Feminist Thought: Knowledge, Consciousness and the Politics of Empowerment. 2nd Edn. London: Routledge.

[B30] Hill Collins P. (2019). Intersectionality as Critical Social Theory. Durham: Duke University Press. 10.1215/9781478007098

[B31] Hughes K. Bellis M. A. Jones L. Wood S. Bates G. Eckley L. (2012). Prevalence and Risk of Violence against Adults with Disabilities: A Systematic Review and Meta-Analysis of Observational Studies. The Lancet 379 (9826), 1621–1629. Available at www.thelancet.com/journals/lancet/article/PIIS0140-6736(11)61851-5/fulltext. 10.1016/s0140-6736(11)61851-5 22377290

[B32] Humphries B. (2008). Social Work Research for Social Justice. Macmillan International Higher Education.

[B33] Jeremiah E. (2006). Motherhood to Mothering and beyond Maternity in Recent Feminist Thought. J. motherhood Initiat. Res. 8 (1/2), 21–33.

[B34] Jones A. Trotman-Jemmott E. (2009). Perceptions of, Attitudes to and Opinions on Child Sexual Abuse in the Eastern Caribbean. Barbados: UNICEF. https://www.unicef.org/infobycountry/files/Child_Sexual_Abuse_in_the_Eastern_Caribbean_Final_9_Nov.pdf.

[B56] Jones A. D. Da Breo H. Trotman Jemmott E. Joseph D. Möller C. (2017). Twenty-one Lessons: Preventing Domestic Violence in the Caribbean. Huddersfield: University of Huddersfield Press.

[B35] Jones A. D. Trotman Jemmott E. Maharaj P. E. Da Breo H. (2014). An Integrated Model for Preventing Child Sexual Abuse: Perspectives from Latin America and the Caribbean. Basingstoke, England: Palgrave Macmillan. 10.1057/9781137377661

[B36] Kempadoo K. Taitt A. (2006). Gender, Sexuality and Implications for HIV/AIDS in the Caribbean: A Review of Literature and Programmes. Bridgetown, Barbados: UNIFEM and IDRC. https://caribbean.unwomen.org/en/materials/publications/2006/6/gender-sexuality-and-hiv-in-the-caribbean.

[B37] Kempadoo K. (2004). Sexing the Caribbean: Gender, Race, and Sexual Labor. New York: Routledge, 272.

[B38] la Riviére Zijdel L. (2004). “Disabled Women and Non–disabled Women, Strategies of Action within the European Context.” Cited in European Parliament 2006. European Parliament Resolution on the Situation of People with Disabilities in the Enlarged European Union: The European Action Plan 2006−2007.

[B39] Le Franc E. Samms-Vaughan M. Hambleton I. Fox K. Brown D. (2008). Interpersonal Violence in Three Caribbean Countries: Barbados, Jamaica, and Trinidad and Tobago. Rev. Panam Salud Publica 24 (6), 409–421. 10.1590/S1020-49892008001200005 19178780

[B40] Luciano D. (2013). Addressing Violence Against Women Living with HIV in Latin America and the Caribbean. Virginia: Rewire. https://rewire.news/article/2013/05/19/addressing-violence-against-women-living-with-hiv-in-latin-america-and-the-caribbean/.

[B41] McMahon S. Huang C-C. Boxer P. Postmus J. L. (2011). The Impact of Emotional and Physical Violence during Pregnancy on Maternal and Child Health at One Year Post-partum. Child. Youth Serv. Rev. 33 (11), 2103–2111. 10.1016/j.childyouth.2011.06.001

[B42] Milletich R. J. Kelley M. L. Doane A. N. Pearson M. R. (2010). Exposure to Interparental Violence and Childhood Physical and Emotional Abuse as Related to Physical Aggression in Undergraduate Dating Relationships. J. Fam. Viol. 25 (7), 627–637. 10.1007/s10896-010-9319-3

[B43] Namy S. Carlson C. O'Hara K. Nakuti J. Bukuluki P. Lwanyaaga J. (2017). Towards a Feminist Understanding of Intersecting Violence against Women and Children in the Family. Soc. Sci. Med. 184, 40–48. 10.1016/j.socscimed.2017.04.042 28501019 PMC5737762

[B44] PAHO/WHO (2007). Perspectives in Health, Washington, DC: PAHO/WHO, 11.

[B45] Schröttle M. Glammeier S. (2013). Intimate Partner Violence against Disabled Women as a Part of Widespread Victimization and Discrimination over the Lifetime: Evidence from a German Representative Study. Int. J. Conflict Violence 7 (2), 232–248. 10.4119/ijcv-3021

[B47] Silverman J. G. Decker M. R. Reed E. Raj A. (2006). Intimate Partner Violence Victimization Prior to and during Pregnancy Among Women Residing in 26 U.S. States: Associations with Maternal and Neonatal Health. Am. J. Obstet. Gynecol. 195 (1), 140–148. 10.1016/j.ajog.2005.12.052 16813751

[B48] Sumi C. Crenshaw K. McCall L. (2013). Toward a Field of Intersectionality Studies: Theory, Applications, and Praxis. Signs: Intersectionality: Theorizing Power Empowering Theor. 38 (4), 785–810. 10.1086/669608

[B49] Taillieu T. L. Brownridge D. A. Tyler K. A. Chan K. L. Tiwari A. Santos S. C. (2015). Pregnancy and Intimate Partner Violence in Canada: A Comparison of Victims Who Were and Were Not Abused during Pregnancy. J. Fam. Violence 31, 567–579. 10.1086/669608

[B50] UNAIDS/WHO (2007). “Situación de la epidemia del SIDA: Informe especial sobre la prevención del VIH.” (Situation of the AIDS Epidemic: A Special Report on HIV Prevention). Geneva: UNAIDS/WHO. www.universia.net.co/HIV/AIDS/view-document/document-648.html.

[B51] UNICEF (2006). Barbados Health and Family Education for Children Survey, Health and Family Education for Children Programme. Barbados: UNICEF Office for the Eastern Caribbean Area.

[B52] UNICEF (2008). Barbados Health and Family Education for Children Survey, Health and Family Education for Children Programme. Barbados: UNICEF Office for the Eastern Caribbean Area.

[B53] UNICEF (2012). Sexual Violence Against Children in the Caribbean: Progress Report 2012. Bridgetown, Barbados: UNICEF Office for the Eastern Caribbean Area.

[B54] United Nation (2007). The United Nations Office on Drugs and Crime and the Latin America and the Caribbean Region of the World Bank. Other Urban Study. https://caribbean.unfpa.org/en/news/gender-thematic-brief.

[B55] World Health Organization (2013). Global and Regional Estimates of Violence against Women: Prevalence and Health Effects of Intimate Partner Violence and Non-partner Sexual Violence. Geneva: World Health Organization.

