# Supplementary figures and images for: Child Sexual Abuse as Lifespan Trauma Within the Context of Intimate Partner Violence: Experiences of Caribbean Women
Source: Front Sociol. 2021 May 11;6:623661. doi: 10.3389/fsoc.2021.623661 (PMC8144712; doi:10.3389/fsoc.2021.623661)

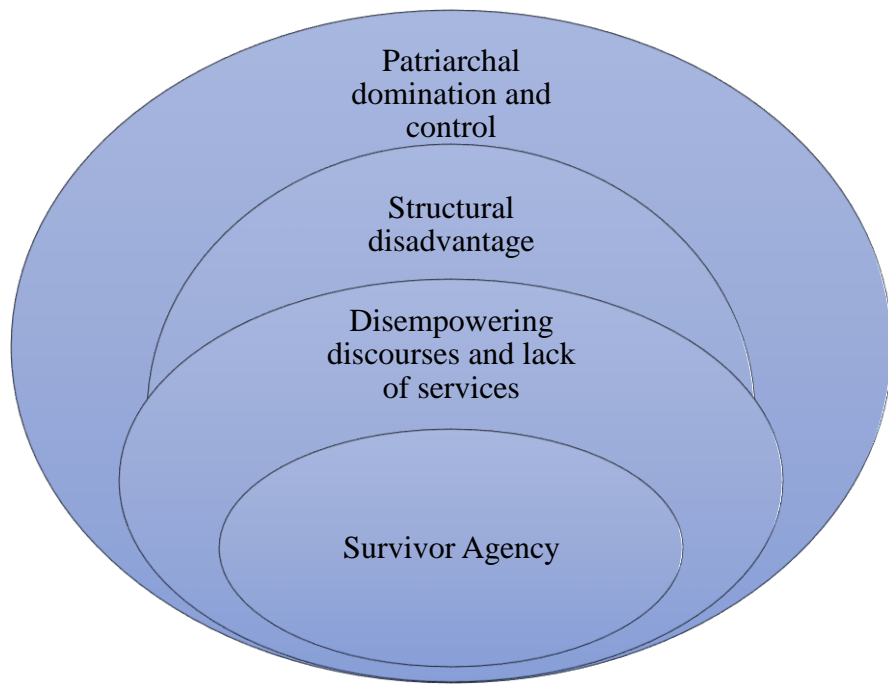

Figure 1 Liminal Survivor Agency

Supplement: Supplementary file 1 [file Image1.pdf]
